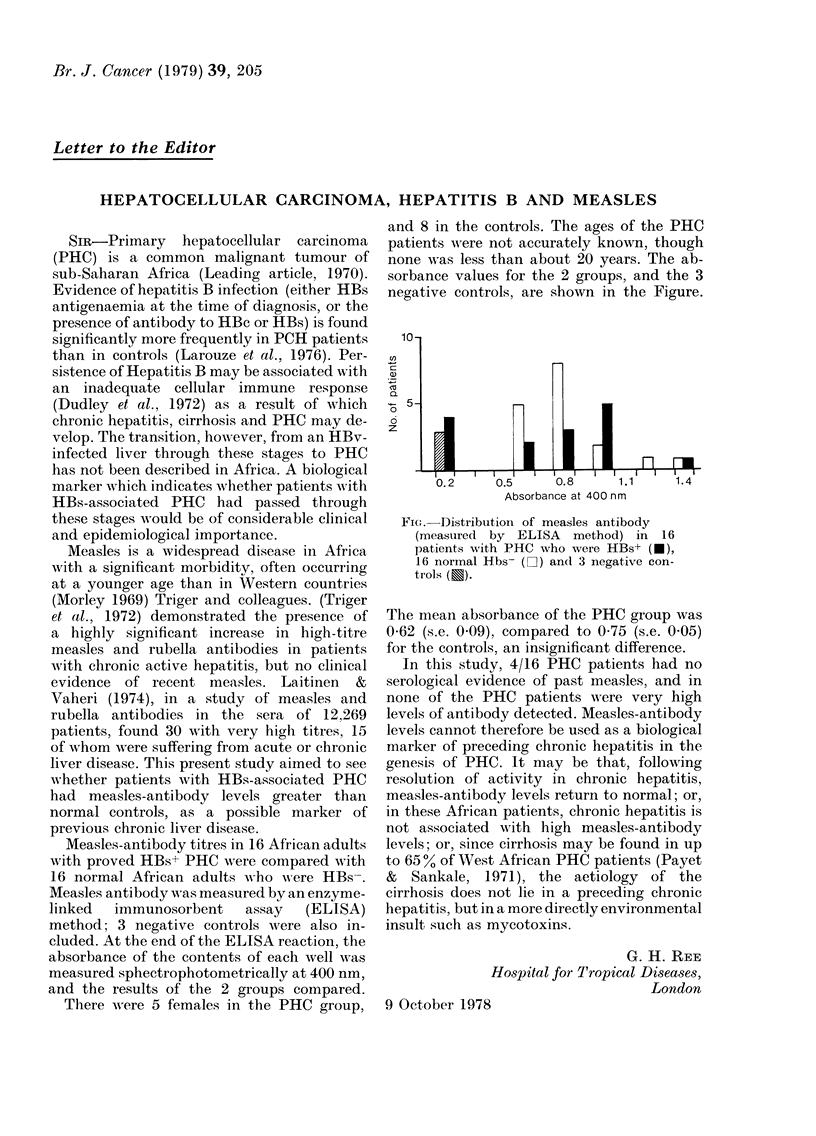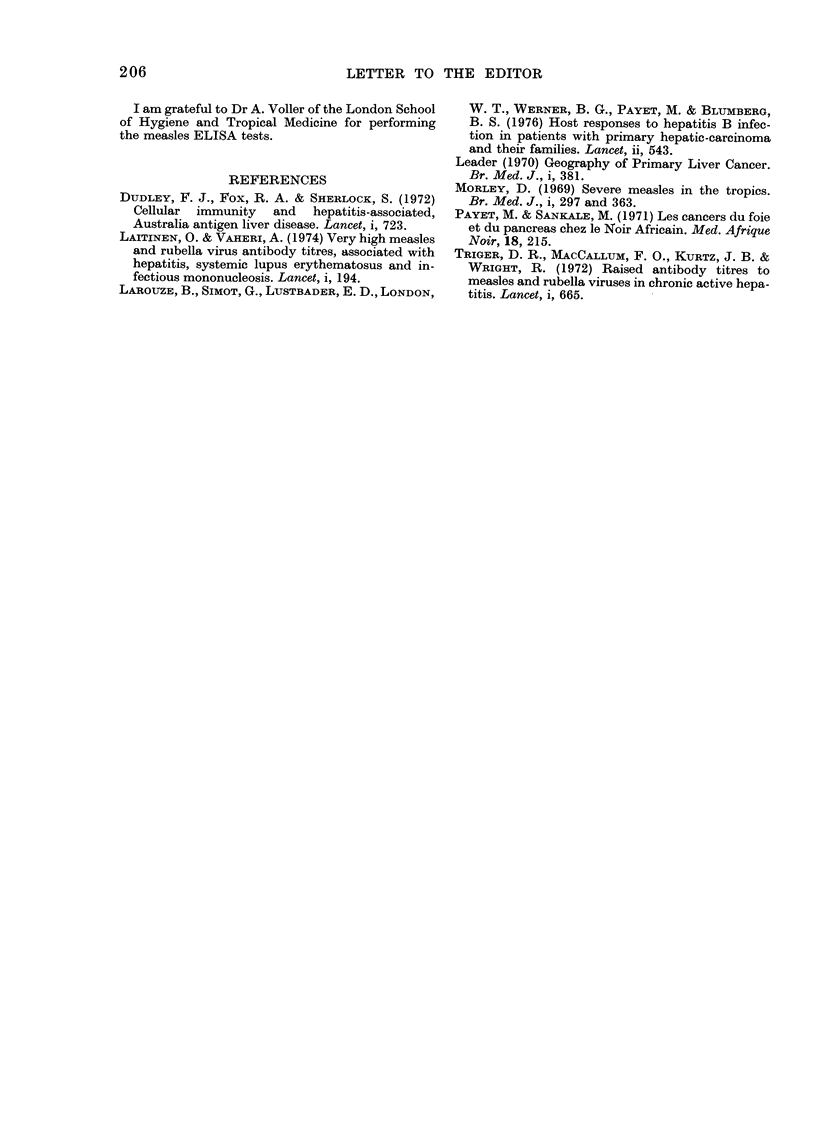# Hepatocellular carcinoma, hepatitis B and measles.

**DOI:** 10.1038/bjc.1979.33

**Published:** 1979-02

**Authors:** G. H. Ree


*Br. J. Cancer* (1979) **39**, 205

### *Letter to the Editor*

### HEPATOCELLULAR CARCINOMA, HEPATITIS B AND MEASLES

SIR—Primary hepatocellular carcinoma (PHC) is a common malignant tumour of sub-Saharan Africa (Leading article, 1970). Evidence of hepatitis B infection (either HBs antigenaemia at the time of diagnosis, or the presence of antibody to HBc or HBs) is found significantly more frequently in PCH patients than in controls (Larouze *et al.*, 1976). Persistence of Hepatitis B may be associated with an inadequate cellular immune response (Dudley *et al.*, 1972) as a result of which chronic hepatitis, cirrhosis and PHC may develop. The transition, however, from an HBv-infected liver through these stages to PHC has not been described in Africa. A biological marker which indicates whether patients with HBs-associated PHC had passed through these stages would be of considerable clinical and epidemiological importance.

Measles is a widespread disease in Africa with a significant morbidity, often occurring at a younger age than in Western countries (Morley 1969) Triger and colleagues. (Triger *et al.*, 1972) demonstrated the presence of a highly significant increase in high-titre measles and rubella antibodies in patients with chronic active hepatitis, but no clinical evidence of recent measles. Laitinen & Vaheri (1974), in a study of measles and rubella antibodies in the sera of 12,269 patients, found 30 with very high titres, 15 of whom were suffering from acute or chronic liver disease. This present study aimed to see whether patients with HBs-associated PHC had measles-antibody levels greater than normal controls, as a possible marker of previous chronic liver disease.

Measles-antibody titres in 16 African adults with proved $HBs^+$ PHC were compared with 16 normal African adults who were $HBs^-$. Measles antibody was measured by an enzyme-linked immunosorbent assay (ELISA) method; 3 negative controls were also included. At the end of the ELISA reaction, the absorbance of the contents of each well was measured sphectrophotometrically at 400 nm, and the results of the 2 groups compared.

There were 5 females in the PHC group, and 8 in the controls. The ages of the PHC patients were not accurately known, though none was less than about 20 years. The absorbance values for the 2 groups, and the 3 negative controls, are shown in the Figure.

FIG.—Distribution of measles antibody (measured by ELISA method) in 16 patients with PHC who were $HBs^+$ (■), 16 normal $Hbs^-$ (□) and 3 negative controls (▨).

The mean absorbance of the PHC group was 0·62 (s.e. 0·09), compared to 0·75 (s.e. 0·05) for the controls, an insignificant difference.

In this study, 4/16 PHC patients had no serological evidence of past measles, and in none of the PHC patients were very high levels of antibody detected. Measles-antibody levels cannot therefore be used as a biological marker of preceding chronic hepatitis in the genesis of PHC. It may be that, following resolution of activity in chronic hepatitis, measles-antibody levels return to normal; or, in these African patients, chronic hepatitis is not associated with high measles-antibody levels; or, since cirrhosis may be found in up to 65% of West African PHC patients (Payet & Sankale, 1971), the aetiology of the cirrhosis does not lie in a preceding chronic hepatitis, but in a more directly environmental insult such as mycotoxins.

G. H. REE
*Hospital for Tropical Diseases, London*

9 October 1978

I am grateful to Dr A. Voller of the London School of Hygiene and Tropical Medicine for performing the measles ELISA tests.

### REFERENCES

Dudley, F. J., Fox, R. A. & Sherlock, S. (1972) Cellular immunity and hepatitis-associated, Australia antigen liver disease. *Lancet*, i, 723.

Laitinen, O. & Vaheri, A. (1974) Very high measles and rubella virus antibody titres, associated with hepatitis, systemic lupus erythematosus and infectious mononucleosis. *Lancet*, i, 194.

Larouze, B., Simot, G., Lustbader, E. D., London, W. T., Werner, B. G., Payet, M. & Blumberg, B. S. (1976) Host responses to hepatitis B infection in patients with primary hepatic-carcinoma and their families. *Lancet*, ii, 543.

Leader (1970) Geography of Primary Liver Cancer. *Br. Med. J.*, i, 381.

Morley, D. (1969) Severe measles in the tropics. *Br. Med. J.*, i, 297 and 363.

Payet, M. & Sankale, M. (1971) Les cancers du foie et du pancreas chez le Noir Africain. *Med. Afrique Noir*, **18**, 215.

Triger, D. R., MacCallum, F. O., Kurtz, J. B. & Wright, R. (1972) Raised antibody titres to measles and rubella viruses in chronic active hepatitis. *Lancet*, i, 665.